# New Avian Influenza Virus (H5N1) in Wild Birds, Qinghai, China

**DOI:** 10.3201/eid1702.100732

**Published:** 2011-02

**Authors:** Yanbing Li, Liling Liu, Yi Zhang, Zhenhua Duan, Guobin Tian, Xianying Zeng, Jianzhong Shi, Licheng Zhang, Hualan Chen

**Affiliations:** Author affiliations: Harbin Veterinary Research Institute, Harbin, People’s Republic of China (Y. Li, L. Liu, Y. Zhang, Z. Duan, G. Tian, X. Zeng, J. Shi, H. Chen);; Qinghai Provincial Center of Animal Disease Control and Prevention, Xining, People’s Republic of China (L. Zhang)

**Keywords:** Avian influenza virus (H5N1), viruses, influenza, wild birds, clade 2.3.2, China, dispatch

## Abstract

Highly pathogenic avian influenza virus (H5N1) (QH09) was isolated from dead wild birds (3 species) in Qinghai, China, during May–June 2009. Phylogenetic and antigenic analyses showed that QH09 was clearly distinguishable from classical clade 2.2 viruses and belonged to clade 2.3.2.

In May 2005, highly pathogenic avian influenza (HPAI) virus (H5N1) caused a disease outbreak in wild birds in the Qinghai Lake region of the People’s Republic of China (*1*). Subsequently, this virus (QH05, clade 2.2) disseminated from Asia to Europe and Africa, which has led to great concern and energetic debates about the role of migratory birds in influenza epidemics (*1**–**5*). In 2006, this virus was detected in migratory birds in Qinghai (*6**,**7*). In 2007, viruses similar to QH05 were isolated from surveyed anseriformes in Qinghai and showed only a short evolutionary distance from earlier viruses (*8*). Genetic diversity of avian influenza viruses (H5N1) was not detected in wild birds in Qinghai before 2008 (*7**,**8*). We report evidence that a second lineage of viruses, in addition to clade 2.2, has emerged in wild birds in Qinghai.

## The Study

**Figure 1:**
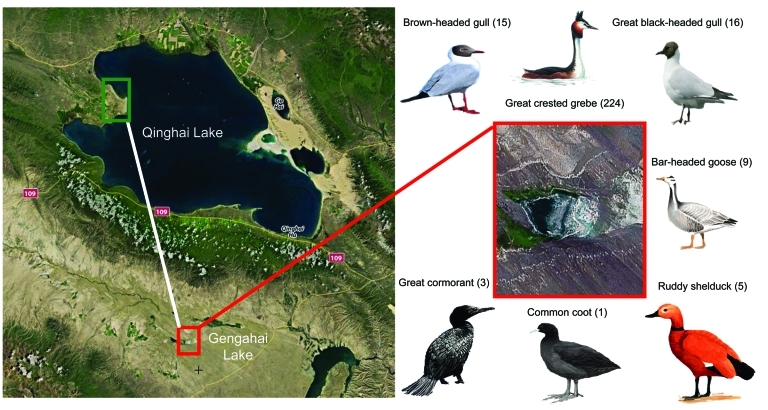
Location in Qinghai, China, of dead birds that were tested for avian influenza virus (H5N1), with images and common names of bird species tested. Red box indicates Gengahai Lake, where dead birds were detected, and green box indicates Bird Islet of Qinghai Lake; the distance between them is 90 km. Numbers of dead birds of each species are indicated in parentheses.

During May 8–June 15, 2009, a total of 273 wild birds died in the wetlands of Gengahai Lake, Qinghai Province, China (Figure 1) during an influenza outbreak. We obtained 224 great crested grebes (*Podiceps cristatus*), 16 great black-headed gulls (*Larus ichthyaetus*), 15 brown-headed gulls (*Larus brunnicephalus*), 9 bar-headed geese (*Anser indicus*), 5 ruddy shelducks (*Tadorna ferruginea*), 3 great cormorants (*Phalacrocorax carbo*), and 1 common coot (family *Rallidae*) (Figure 1).

To determine the pathogenesis of this outbreak, we obtained organs, including lung and brain, and cloacal swabs from 13 birds at different times. Tissue samples were inoculated into 10-day-old, embryonated, specific pathogen–free eggs for virus isolation. Hemagglutinin and neuraminidase subtypes were determined as described (*9*). Eleven avian influenza viruses (H5N1) were isolated from 3 species of wild birds: 4 from great crested grebes, 5 from great black-headed gulls, and 2 from brown-headed gulls. Results of virus isolation for samples from a bar-headed goose and a shelduck were negative. Samples from great cormorants and a common coot were not obtained and tested.

We sequenced genomes of the 11 viruses and found that the viruses (QH09) were closely related and showed 100% homology at the nucleotide level. Sequences of the entire genome of QH09 (representative strain A/great crested grebe/Qinghai/1/09) are available in GenBank (accession nos. CY063315–CY063322).

Hemagglutinin of QH09 virus had a series of basic amino acids (PQRERRRKR) at the cleavage site. Neuraminidase of this virus had a deletion of 20 aa at residues 49–68 in the stalk region. No amino acid substitutions conferred resistance to adamantane or neuraminidase inhibitors. Unlike many isolates related to Qinghai Lake strains of clade 2.2, QH09 virus does not have an E627K substitution in basic polymerase. Nonstructural protein 1 had a deletion of 5 aa at residues 80–84, which is commonly observed in HPAI viruses (H5N1) that are circulating in Southeast Asia.

Seven gene segments of QH09, except for the acidic polymerase (PA) gene, showed the greatest homology with 2 clade 2.3.2 viruses, A/little egret/Hongkong/8863/07(H5N1) and A/whooper swan/Hokkaido/1/08(H5N1); identities were 97.0%–99.5%. The strain that showed the greatest homology with QH09 PA was A/chicken/Yamaguchi/7/04 (H5N1), which was the precursor of the PA gene of the A/bar-headed goose/Qinghai/2/05 virus (*1*) (Table). Homology of the 8 gene segments of QH09 with QH05-like viruses was 92%–96.3%.

**Table:** Percentage homology of influenza viruses closely related to avian influenza virus (H5N1) QH09, Qinghai, China*

Gene	Virus	% Homology
HA	LE/Hong Kong/8863/07	97.1
NA	WS/Hokkaido/1/08	98.9
PB2	LE/Hong Kong/8863/07	98.7
PB1	WS/Hokkaido/1/08	98.8
PA	CK/Yamaguchi/7/04	97.2
NP	WS/Hokkaido/1/08	98.3
M	LE/Hong Kong/8883/07	98.9
NS	LE/Hong Kong/8863/07	99.5

*HA, hemagglutinin; NA, neuramindase; PB, basic polymerase; PA, acidic polymerase; NP, nucleoprotein; M, matrix; NS, nonstructural.

**Figure 2:**
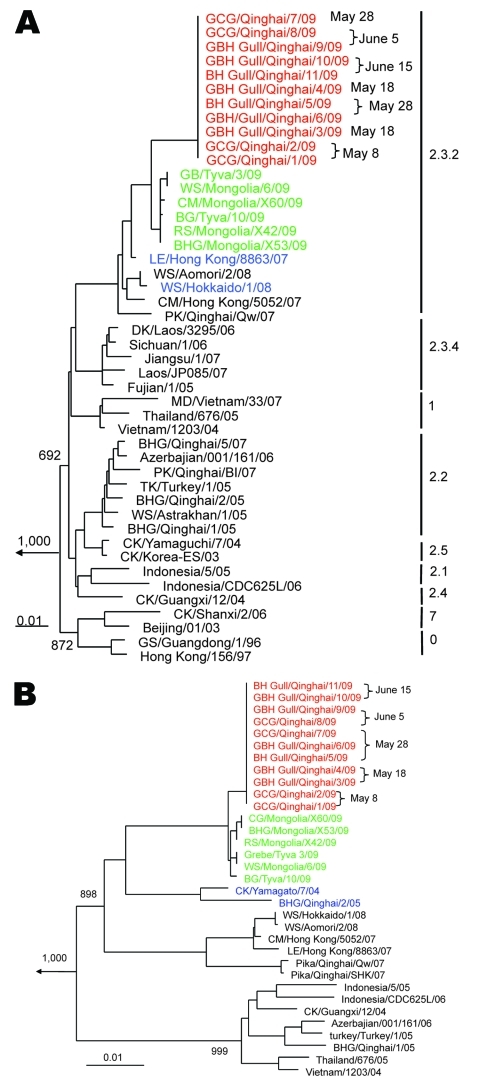
Phylogenetic trees of hemagglutinin genes (nt 29–1,728) (A) and acidic polymerase genes (nt 25–2,151) of avian influenza viruses (H5N1) (B). Clade numbers are indicated on the right in panel A. Trees were constructed by using the PHYLIP program of ClustalX software version 1.81 (www.clustal.org), the neighbor-joining algorithm, and rooted to A/chicken/Pennsylvania/1/83(H5N2). Bootstrap analysis was performed with 1,000 replications. Viruses obtained in this study are shown in red, previously detected viruses that are closely related to avian influenza virus (H5N1) QH09 are shown in blue, and closely related viruses that were detected after the Qinghai wild bird outbreak in 2009 are shown in green. Dates of virus isolation are shown. Scale bars indicate nucleotide substitutions per site. GCG, great crested grebe; GBH, great black-headed gull; BH, brown-headed gull; GB, grebe; WS, whooper swan; CM, common magpie; BG, bean goose; RS, ruddy shelduck; BHG, bar-headed goose; LE, little egret; PK, pike; DK, duck; MD, Muscovy duck; TK, turkey; CK, chicken; GS, goose; CG, common goldendye.

Phylogenetic analysis showed that 7 of 8 gene fragments of QH09, except for the PA gene, mapped with clade 2.3.2 viruses A/little egret/Hongkong/8863/07 and A/whooper swan/Hokkaido/1/08 and were in a different cluster than clade 2.2 viruses isolated in Qinghai during 2005–2007 (Figure 2, panel A). The PA gene of QH09 had the same lineage as A/chicken/Yamaguchi/7/04 (Figure 2, panel B).

Antigenic analyses by hemagglutination inhibition (HI) assay with chicken antiserum against a QH05 virus (A/bar-headed goose/Qinghai/3/05) (clade 2.2) and QH09 virus showed that the cross-reactive HI titer of QH09 virus (64) was 16-fold lower than that of homologous QH05 virus (1,024). The cross-reactive HI titer of QH05 virus (128) was also 16-fold lower than that of homologous QH09 virus (2,048).

## Conclusions

Genetic and antigenic characterization of HPAI viruses (H5N1) from wild birds in Qinghai in 2009 suggests that these viruses are closely related to clade 2.3.2 and are clearly distinguishable from the classical QH05 clade 2.2. Previously reported QH05-like virus represented >4 genotypes, and no 2 QH05-like viruses were identical at the amino acid or nucleotide sequence levels (*1**,**6*). Although isolates of QH09 were obtained from 3 species at different times, 100% homology of the 11 isolates of QH09 was observed, which suggests that wild birds in Qinghai in 2009 were newly infected by 1 strain of virus.

Clade 2.3.2 viruses from wild nonpasserine bird species were reported in Hong Kong in 2007–2008 (*10*). The fact that a similar virus was isolated from a whooper swan (order Anseriformes) in Japan in 2008 showed that clade 2.3.2 was dispersed by migration of wild birds (*11*). Our results indicated that QH09 virus is a reassortant containing 7 gene segments of clade 2.3.2 viruses detected in wild birds and the PA gene of CK/Yamaguchi/7/04-like virus, which contributed the PA gene to 1 QH05 virus (*1*).

Similar genotypes of QH09-like clade 2.3.2 viruses were also detected in great-crested grebes and black-headed gulls in Russia in 2009 (*12*). Bar-headed geese, whooper swans, and other anseriforme birds in Mongolia were infected with QH09-like clade 2.3.2 viruses (*13*). Therefore, QH09-like clade 2.3.2 virus is likely adapted to wild birds and is similar to clade 2.2 viruses, and its presence in Qinghai suggests that wild birds have spread this virus to other regions.

The possibility that wild birds in Qinghai in 2009 were infected by domestic fowl that harbored clade 2.3.2 virus is low because of the location of Qinghai Province and the scarcity of poultry in this region. In addition, no influenza outbreak in poultry occurred in this region in 2009. Therefore, it is likely that wild birds spread the virus.

How wild birds transmit HPAI virus (H5N1) is not clear. The ecology, epidemiology, genetics, and evolution of this virus are not fully understood. QH05-like clade 2.2 viruses and clade 2.3.2 viruses have been detected in wild pikas (*14*). However, whether the 2 virus clades have propagated in other mammalian hosts is unknown.

Qinghai Lake is located near multiple avian flyways. Although there are no reports of detection of clade 2.3.2 virus in wild birds near Qinghai Lake, the finding of clade 2.3.2 virus in the Gengahai wetlands of Qinghai increases concerns about a potential pandemic and the likelihood that avian influenza virus (H5N1) will again spread and increase its genetic diversity. Therefore, determining movements of wild migratory waterfowl from Qinghai Lake and their virologic status is needed to assess potential avian vectors of HPAI virus (H5N1).
